# Supplementary material for: NFκB Inhibition Mitigates Serum Amyloid A-Induced Pro-Atherogenic Responses in Endothelial Cells and Leukocyte Adhesion and Adverse Changes to Endothelium Function in Isolated Aorta
Source: Int J Mol Sci. 2018 Dec 28;20(1):105. doi: 10.3390/ijms20010105 (PMC6337750; doi:10.3390/ijms20010105)
Supplement: Supplementary file 1 [file ijms-20-00105-s001.pdf]

NFκB Inhibition Mitigates Serum Amyloid A-Induced Pro-Atherogenic Responses in Endothelial Cells and Leukocyte Adhesion and Adverse Changes to Endothelium Function in Isolated Aorta

Abigail Vallejo [1], Belal Chami [1], Joanne M. Dennis [1], Martin Simone [1] Gulfam Ahmad [1], Adrian I. Abdo [2], Arpeeta Sharma [3], Waled A Shihata,[3,4,5] Nathan Martin [1], Jaye P.F. Chin-Dusting [3,4,5], Judy B. de Haan [3,6,7] and Paul K. Witting[1*]

[1] Discipline of Pathology, Sydney Medical School, The University of Sydney, NSW, 2006, Australia; aval3972@uni.sydney.edu.au (A.V.); belal.chami@sydney.edu.au (B.C.); joanne.dennis@sydney.edu.au (J.M.D.); msim6013@uni.sydney.edu.au (M.S.); gulfam.ahmad@sydney.edu.au (G.A.); nmar7397@uni.sydney.edu.au (N.M.);

[2] Heart Research Institute, Newton, NSW, 2053, Australia; adrian.abdo@hri.org.au

[3] Baker Heart and Diabetes Institute, Victoria, Australia; Arpeeta.sharma@baker.edu.au (A.S.); jaye.chin-dusting@baker.edu.au (J.P.F.C.-D.); judy.dehaan@baker.edu.au (J.B.D.H.)

[4] Department of Medicine, Monash University, Victoria, 3500 Australia; waled.shihata@monash.com.au (W.A.S.)

[5] Cardiovascular Disease Program, Biomedicine Discovery Institute, Monash University [£]Department of Pharmacology, Monash University, Victoria, 3800, Australia

[6] Department of Immunology, Monash University, Victoria, 3004, Australia.

[7] Department of Physiology, Anatomy & Microbiology, School of Life Sciences, La Trobe University, 3083, Australia.

Supplementary data

Table S1. HCtAE cell confluency as determined by imaging with an IncuCyte system.

Well	Treatment group	Confluency (%)
A1	1.1 Control	56.85298
B1	1.2 Control	56.13226
A2	1.1 SAA	59.34018
B2	1.2 SAA	64.7347
A3	1.1 BAY11	33.76793
B3	1.2 BAY11	35.37162

[a] High-definition D phase-contrast images were acquired for each well in a 6-well plate using the The IncuCyte Zoom® live cell imaging system (Essen BioScience, Australia). The system software calculated the average confluency of each individual field imaged to obtain the overall mean (n=4) level of confluency for the well expressed as a percentage of the total area imaged (%). Levels of confluency were then used to normalise total secretary IL-6 as determined by ELISA.

Figure S1. Gene sequencing

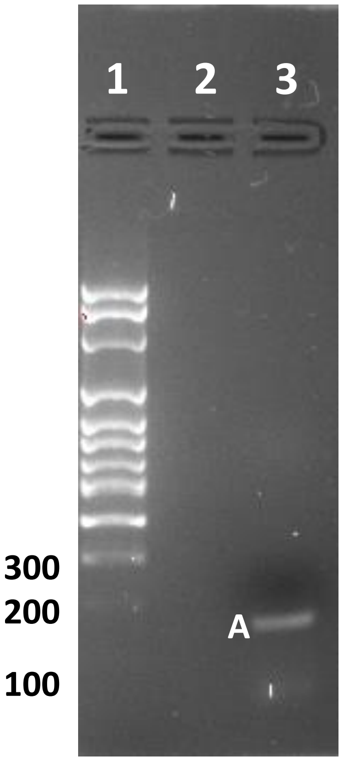

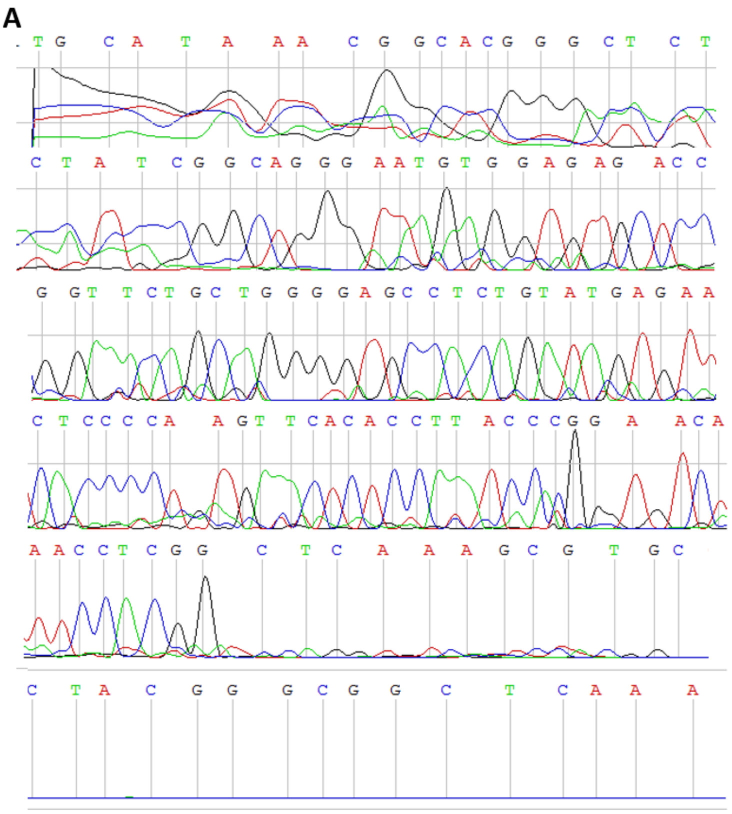

Figure S1(i). RT-PCR gel for TF expression

Lane 1. Hyperladder 100 Bp Plus

Lane 2. No sample

Lane 3. HCtAE cDNA

Note Band A corresponds to an amplification product of 157bp

Figure S1(ii). Sequencing chromotagram for purified bands (Gentle Software)

DNA from bands noted as *A* in Figure 1 were extracted from agarose gel, purified and sequenced. Figure shows good quality chromatography data for reliable sequence data.

Experimentally determined Sequence

TGCATAAACGGCACGGGCTCTCTATCGGCAGGGAATGTGGAGAGACCGGTTCTGCT
GGGGAGCCTCTGTATGAGAACTCCCCAAGTTCACACCTTACCCGGAACAAACCTCGG
CTCAAAGCGTGCCTACGGGCGGCTCAAATTATTGCATCTTTGCTGGGGGTCCCCCGG
CGGGGGGGGGGGAGGGCCAAAAGGAAATATTGCACG

Figure S1(iii). Tissue factor sequencing result

Homo sapiens coagulation factor III (thromboplastin, tissue factor) mRNA, complete cds

Sequence ID: BT019808.1 **Length:** 888 **Number of Matches:** 1

▶ See 1 more title(s)

Range 1: 312 to 422 GenBank Graphics ▼ Next Match ▲ Previous Match

Score	Expect	Identities	Gaps	Strand
148 bits(80)	3e-32	102/111(92%)	7/111(6%)	Plus/Plus

```
Query  10   GGCACGGG-C-TCT-CTA-TCGGCAGGGAATGTGGAGAG-ACCGGTTCTGCTGGGGAGCC  64
            |||||||| | ||| |||  |||||||||||||||||| ||||||||||||||||||||
Sbjct  312  GGCACGGGTCTTCTCCTACCCGGCAGGGAATGTGGAGAGCACCGGTTCTGCTGGGGAGCC  371

Query  65   TCTGTATGAGAACTCCCCA-AGTTCACACCTTACCCGGA-ACAAACCTCGG  113
            ||||||||||||||||||| ||||||||||||||| ||| |||||||||||
Sbjct  372  TCTGTATGAGAACTCCCCAGAGTTCACACCTTACCTGGAGACAAACCTCGG  422
```

Figure S1(iv). Band A NCBI Nucleotide Blast result

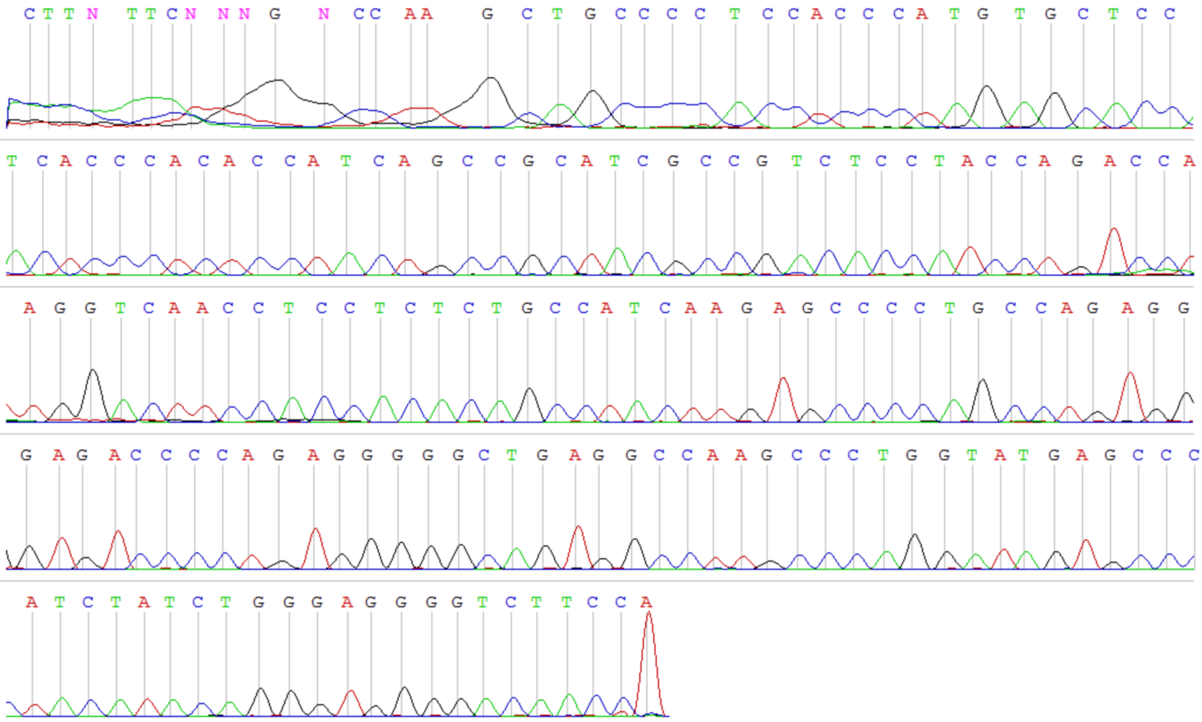

Figure S1(i-iv). Sequencing Chromatogram for purified bands (Gentle Software)

DNA from bands noted *A* Figure 1 were extracted from agarose gel, purified and sequenced. Figure shows good quality chromatography data for reliable sequence data. Percentage match obtained was 92% identity.

Figure S2(i). Beta actin house keeping gene

Experimentally determined Sequence

TGGTCTCATATTCATTGAAGGAGCGAAGGTGTCTCGTGGATGCCCAGGACCCTGCCA
GGAAGGAAGGCTGGAAAGTGCCTAGGACACCGGACCGCTATTGCCAATGGTGATGA
CCTGGCCGTCAGGCAGCTCGTACTCTTCTCCAGAGAAGAGCAGGAAGCAGCCGTGG
CCATTTGGCTCGAATCAGGTGT

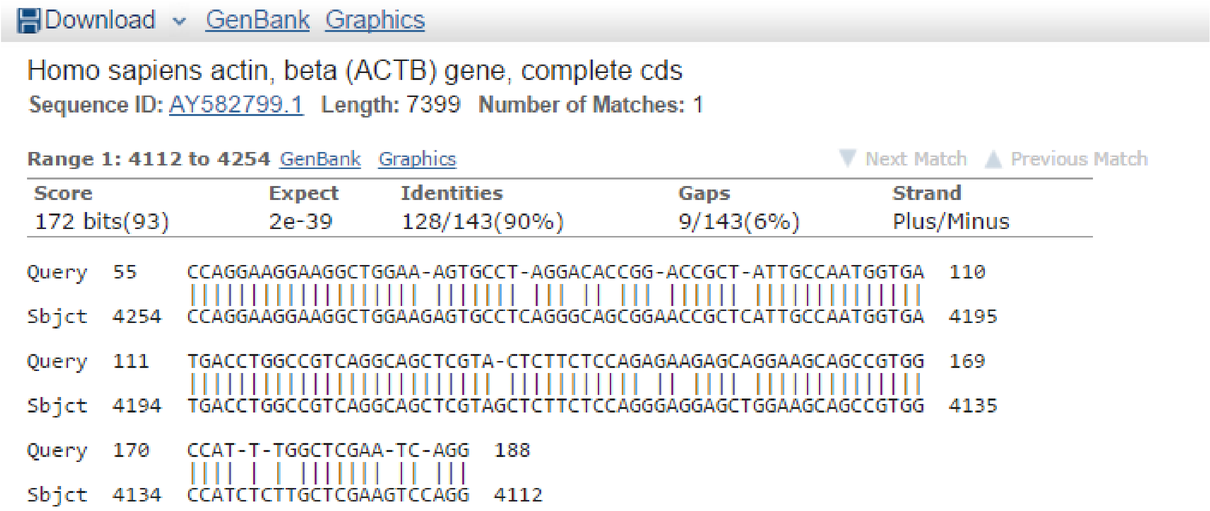

Figure S2(ii). Beta actin Nucleotide Blast

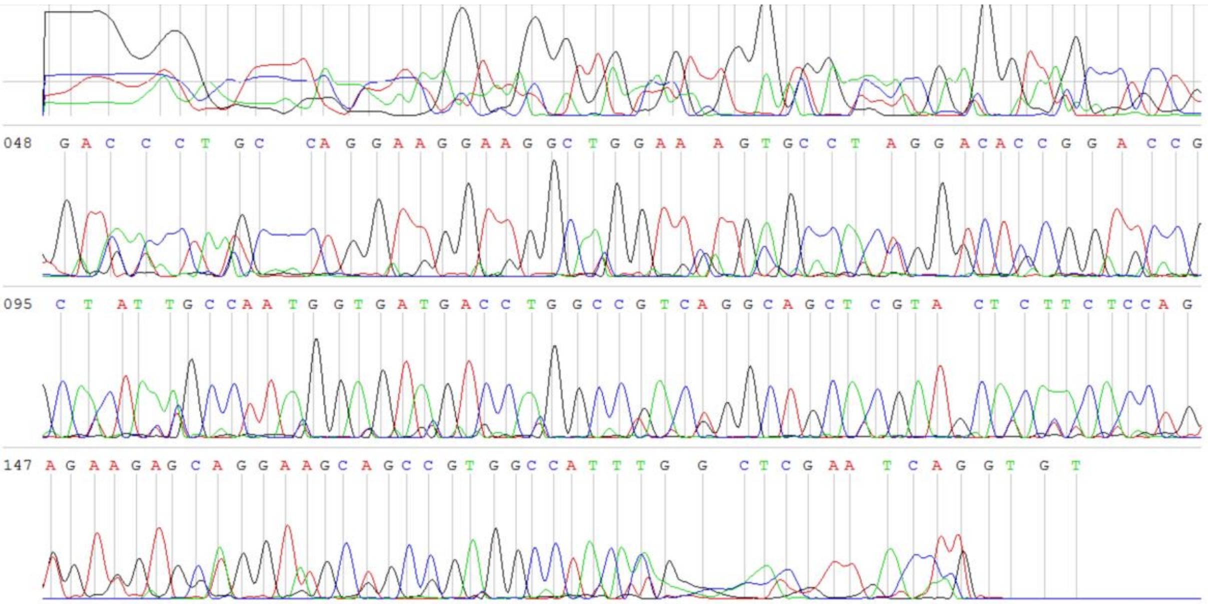

Figure S2(iii). Beta actin Sequencing Chromatogram

DNA from a single band was extracted from agarose gel (as per figure S2(i) above), purified and sequenced. Figure shows good quality chromatography data for reliable sequence data. Percentage match obtained was 90% identity.

Figure S3(i). TNF gene sequencing

Experimentally determined sequence

CTTNTTCNNNGNCCAAGCTGCCCCTCCACCCATGTGCTCCTCACCCACACCATCAGC
CGCATCGCCGTCTCCTACCAGACCAAGGTCAACCTCCTCTCTGCCATCAAGAGCCCC
TGCCAGAGGGAGACCCCAGAGGGGGCTGAGGCCAAGCCCTGGTATGAGCCCATCTA
TCTGGGAGGGGTCTTCCA

Homo sapiens isolate RSH TNF gene, complete cds

Sequence ID: KY500678.2 **Length:** 1796 **Number of Matches:** 1

Range 1: 1520 to 1696 GenBank Graphics ▼ Next Match ▲ Previous Match

Score	Expect	Identities	Gaps	Strand
320 bits(173)	9e-84	176/177(99%)	1/177(0%)	Plus/Plus

```
Query  13    CCAA-GCTGCCCCTCCACCCATGTGCTCCTCACCCACACCATCAGCCGCATCGCCGTCTC  71
             |||| |||||||||||||||||||||||||||||||||||||||||||||||||||||||
Sbjct  1520  CCAAGGCTGCCCCTCCACCCATGTGCTCCTCACCCACACCATCAGCCGCATCGCCGTCTC  1579

Query  72    CTACCAGACCAAGGTCAACCTCCTCTCTGCCATCAAGAGCCCCTGCCAGAGGGAGACCCC  131
             ||||||||||||||||||||||||||||||||||||||||||||||||||||||||||||
Sbjct  1580  CTACCAGACCAAGGTCAACCTCCTCTCTGCCATCAAGAGCCCCTGCCAGAGGGAGACCCC  1639

Query  132   AGAGGGGGCTGAGGCCAAGCCCTGGTATGAGCCCATCTATCTGGGAGGGGTCTTCCA  188
             |||||||||||||||||||||||||||||||||||||||||||||||||||||||||
Sbjct  1640  AGAGGGGGCTGAGGCCAAGCCCTGGTATGAGCCCATCTATCTGGGAGGGGTCTTCCA  1696
```

Figure S3(ii). TNF Nucleotide Blast

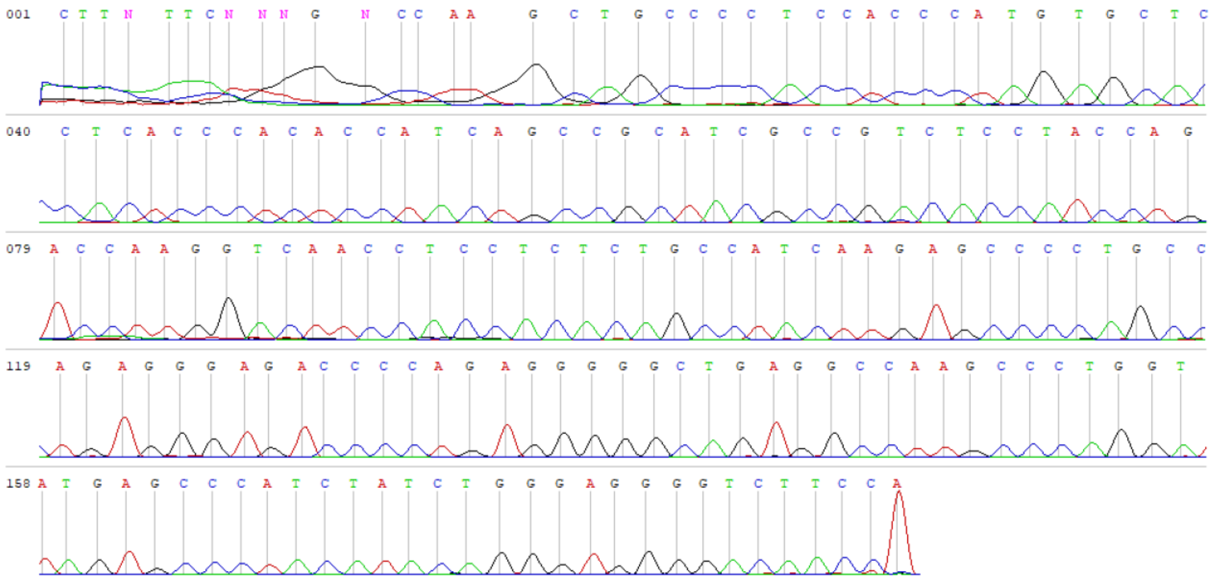

Figure S3(iii). TNF Sequencing Chromatogram

DNA from a single band was extracted from agarose gel (as per figure S2(i) above), purified and sequenced. Figure shows good quality chromatography data for reliable sequence data. Percentage match obtained was 99% identity.

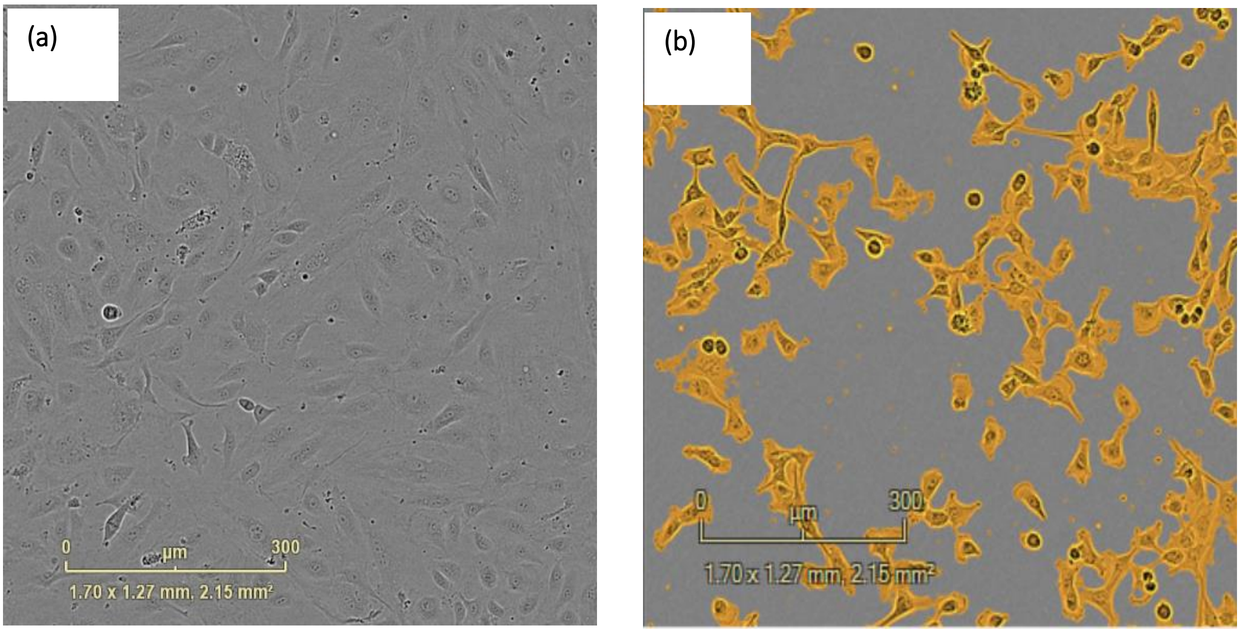

Figure S4. Cultured HCtAE cell confluence as assessed with an IncuCyte imaging system.

The IncuCyte Zoom® live cell imaging system (Essen BioScience, Australia) was used to measure the level of cell confluency (expressed as a percentage of the total area imaged) immediately prior to the time of harvest. (a) Representative field image of well A1 (*Table S1*); showing areal coverage at the 56% confluency level. (b) Representative field image of well B3 (*Table S1*) showing fewer cells in the same areal field (35%) – orange highlight shows confluence mask employed by the software to identify cells). Images were taken using a fixed 10*x* objective lens (Nikon, Australia).
